# Microsatellite instability in the smoldering form of adult T-cell leukemia/lymphoma (ATL) in Brazil

**DOI:** 10.1186/1742-4690-8-S1-A148

**Published:** 2011-06-06

**Authors:** Marcelo Magalhães, Lourdes Farre, Achilea Bittencourt

**Affiliations:** 1 Laboratory of Experimental Pathology, CPQGM, FIOCRUZ, Salvador, Bahia, 40296710, Brazil; 2 Department of Pathology, HUPES, Federal University of Bahia, Salvador, Bahia, 40296710, Brazil

## Background

Microsatellites are short, repetitive elements in genomic DNA that are nonrandomly distributed throughout the human genome. Microsatellite instability (MSI) originates from the systematic accumulation of uncorrected deletion/insertion in repetitive DNA tracts in cancer cells with a deficient mismatch repair system. In ATL, the presence of MSI has been shown in aggressive types, principally in acute ATL. However, few studies have evaluated MSI in smoldering ATL. The aim of this study was to investigate MSI in patients from Bahia (Brazil) with ATL, including the smoldering subtypes.

## Material and methods

Sixteen patients with ATL were investigated: four cases of acute ATL, three patients with chronic ATL, two with lymphoma and seven with the smoldering type. DNA was extracted from peripheral blood mononuclear cells (PBMC) and corresponding control DNA was obtained from the oral mucosa and/or nails of each patient. The D10S190, D10S191, D11S1391 and DCC microsatellite markers were amplified by PCR using primers labeled with FAM, VIC, NET and PET and analyzed using Peak Scanner Software (Applied Biosystems).

## Results

Three of the sixteen patients (18%) had MSI in one locus. Two of these patients had smoldering ATL and one had the lymphoma type. In one patient with acute ATL, MSI was observed in two markers and was classified as a replication error (RER+) phenotype.

## Conclusions

MSI may be found in smoldering ATL. MSI may be associated with pathogenesis of ATL.

